# An integrative perspective on the interplay between early maladaptive schemas and mental health: The role of self‐compassion and emotion regulation

**DOI:** 10.1002/jclp.22755

**Published:** 2019-02-08

**Authors:** Duygu Yakın, Tülin Gençöz, Laura Steenbergen, Arnoud Arntz

**Affiliations:** ^1^ Department of Psychology, Istanbul Arel University Istanbul Turkey; ^2^ Department of Clinical Psychology, University of Amsterdam Amsterdam The Netherlands; ^3^ Department of Psychology, Middle East Technical University Ankara Turkey; ^4^ Cognitive Psychology Unit, Institute for Psychological Research & Leiden Institute for Brain and Cognition, Leiden University Leiden The Netherlands

**Keywords:** difficulties in emotion regulation, early maladaptive schemas, life satisfaction, psychopathology, self‐compassion

## Abstract

**Objectives:**

We aimed to test whether negative emotion regulation difficulties and self‐compassion mediate the relationship between early maladaptive schemas (EMSs) and symptoms of psychopathology and life satisfaction.

**Methods:**

Data were collected from 296 adults (179 females, 117 males), whose age ranged from 17 to 52 years. The mediating roles of self‐compassion and negative emotion regulation were examined via Hayes’ procedure (PROCESS) for multiple mediation.

**Results:**

Negative emotion regulation was the only mediator to psychopathological symptoms, with no additional role for self‐compassion, whereas self‐compassion mediated only to life satisfaction, with no additional role for negative emotion regulation.

**Conclusions:**

The results provide evidence for unique mediating roles of negative emotion regulation and self‐compassion, depending on the outcome variable. That helps to understand how problems that may be identified in terms of positive and negative domains are related to EMSs, and allows to put forward potential strategies within the frame of schema therapy.

## 1 INTRODUCTION

Contemporary psychology trends define mental health as a state of well‐being that involves positive and negative affective dimensions (Kahl, Winter, & Schweiger, 2012); for full mental health, the presence of positive emotions is *as important as* the absence of negative emotions and skills to regulate both positive and negative emotions (Gratz & Roemer, 2004; Keyes, 2005; Westerhof & Keyes, 2010). Hence, to understand mental health, one needs to consider both the absence of possibly negatively contributing factors (e.g., psychopathological symptoms) and the presence of possibly positively contributing factors (e.g., life satisfaction).

As an attempt to understand the complex dynamics of psychopathological states, such as personality disorders, increasing attention has been drawn toward the schema theory. According to schema theory, early maladaptive schemas (EMSs) are essential building blocks for a person’s personality, with EMSs being “developed during childhood and elaborated throughout one’s lifetime, and dysfunctional to a significant degree” (Young, Klosko, & Weishaar, 2003, p. 7). Young et al. (2003) listed five primary emotional childhood needs: (a) safety and nurturance (including secure attachment), (b) autonomy, competence, and the sense of identity, (c) freedom to express needs, emotions, and opinions, (d) spontaneity and play, (e) realistic limits and self‐control. When these fundamental emotional needs are violated, EMSs are formed. Notably, EMSs have been found to be positively related to a number of psychopathological states, such as mood and anxiety disorders (Hawke & Provencher, 2011), eating disorders (Pugh, 2015), chronic pain (Voderholzer et al., 2014), substance dependence (Shorey, Stuart, & Anderson, 2014), and personality disorders (Bamelis, Evers, Spinhoven, & Arntz, 2014; Giesen‐Bloo et al., 2006). Other research has documented a relationship of EMSs with a wide range of variables like lower satisfaction with life (Sahraee, Yusefnejad, & Khosravi, 2011), higher interpersonal conflicts (Messman‐Moore & Coates, 2007), lower marital satisfaction (Yoosefi, Etemadi, Bahrami, Fatehizade, & Ahmadi, 2010), and maladaptive self‐defeating humor (Dozois, Martin, & Bieling, 2009). However, research on how EMSs contribute to the onset and development of such psychopathologies and lower life satisfaction, and which factors may be influential, is scarce.

To study the development of EMSs, attempts have been made to validate theoretically meaningful schema domains associated with different childhood needs. Despite inconsistencies, domains have been put forward (Cecero, Nelson, & Gillie, 2004; Hoffart et al., 2006; Kriston, Schäfer, von Wolff, Härter, & Hölzel, 2012; Lee, Taylor, & Dunn, 1999; Rijkeboer & Van Den Bergh, 2006; Saritaş & Gençöz, 2011; Schmidt, Joiner, Young, & Telch, 1995; Soygüt, Karaosmanoğlu, & Cakir, 2009; Young 1990/1999). One of the dimensions (i.e., domains) put forward as such, is the disconnection/rejection (D/R) domain, that is, characterized by problems regarding attachment stability. This domain contains EMSs such as abandonment/instability, mistrust/abuse, emotional deprivation, defectiveness/shame, and social isolation/alienation. Individuals who are displaying high scores on this D/R domain lack feeling of acceptance, nurturance, stability, and belonging as children. Due to its association with a variety of psychological problems (Bosmans, Braet, & Van Vlierberghe, 2010; Roemmele & Messman‐Moore, 2011; Specht, Chapman, & Cellucci, 2009), this domain is considered the most important. Also, compared with other domains, the D/R domain seems to display a more reliable structure across studies (Kriston et al., 2012). Therefore, following Hawke and Provencher (2011), we select EMSs based on theoretical relevance and focus on EMSs that are relevant to the D/R domain for the current study.

### 1.1 The role of emotion regulation in schema theory

Consistent with the main focus of schema therapy (S. H. Kellogg & Young, 2006), difficulties to regulate negative, as opposed to positive, emotions, are closely associated to psychopathologies and lower satisfaction with life; hence, according to Werner and Gross (2010), emotion regulation is the first step in planning an effective treatment strategy. Based on the four essential steps toward adaptive emotion regulation (Gratz & Roemer, 2004), Dadomo et al. (2016) identified a schema therapy model of emotion regulation and suggested relational and experiential techniques for adaptive regulation of emotions. Despite schema therapy not addressing emotion regulation directly, schema therapy treatment techniques (indirectly) focus on the regulation of negative emotions via systematic interventions (Arntz & Weertman, 1999; Fassbinder, Schweiger, Martius, Brand‐de Wilde, & Arntz, 2016; S. Kellogg, 2012; S. H. Kellogg & Young, 2006).

Emotion regulation is generally viewed as referring to cognitive and behavioral actions that aim at resisting to be carried away by strong emotions and at responding appropriately given the situation (Gratz & Roemer, 2004; Koole, 2009). However, according to Gratz and Roemer (2004), emotion regulation is not restricted to diminishing emotions; awareness and acceptance of emotions are essential parts of the emotion regulation process (Gratz & Roemer, 2004). *Emotion dysregulation* refers to an impaired ability to flexibly regulate and manage emotional reactions, which often leads to psychopathological symptoms (Gratz & Roemer, 2004). Interestingly, both emotion dysregulation and the development of EMSs are the results of childhood maltreatment (Dvir, Ford, Hill, & Frazier, 2014; Rellini, Vujanovic, Gilbert, & Zvolensky, 2012). Regarding the link between emotion regulation and EMSs, higher levels of EMSs are related to more emotion dysregulation (Young et al., 2003). More specifically, Masomi, Hejazi, and Sobhi (2014) postulated that the emotional responses of individuals are determined according to the rules drawn by the EMSs, as such contributing to psychopathology. That is, EMSs and poor EMS coping skills (i.e., how one responds to emotions triggered by the activation of an EMS) might give rise to strong negative emotions, subsequent psychopathology, and hence, lower satisfaction with life. However, the role of emotion regulation in the relationship between EMSs and lower life satisfaction has, to the best of our knowledge, not been addressed empirically.

### 1.2 Self‐compassion as a form of emotion regulation

Psychological well‐being is associated with an individual’s ability to cope with adversity rather than with distress caused by adverse childhood experiences (Keyfitz, Lumley, Hennig, & Dozois, 2013). One such coping mechanism is self‐compassion; soothing oneself with warmth, acceptance, and care. According to Neff (2003a), self‐compassion involves three bipolar continuums, referring to an individual’s ability to (a) treat oneself with gentleness and acceptance rather than criticism and belittling (i.e., self‐kindness vs. self‐judgment), (b) acknowledge failures or imperfections as common human experiences rather than unique and isolated to the individual (i.e., common humanity vs. isolation), and (c) find balance between nonjudgmental appraisal and the suppression of emotions rather than pessimistic self‐victimization (i.e., mindfulness vs. overidentification). As such, self‐compassion enables an individual to handle emotions more adaptively rather than struggling with self‐criticism, self‐blame, and irrational thoughts. Next to that, self‐compassion provides a safe emotional distance from adverse events and a more objective stand, enabling the individual to gain a more realistic understanding of the event (Finlay‐Jones, Rees, & Kane, 2015). Hence, whereas the increased severity of psychopathological symptoms mainly involves failing to regulate negative emotions, self‐compassion focuses on the positive psychological strength that fuels resiliency (Fredrickson, 2001; Gratz & Roemer, 2004). Importantly, self‐compassion does not replace negative emotions with positive ones but facilitates positive emotions by embracing negative ones (Barlow, Goldsmith Turow, & Gerhart, 2017; Neff, 2003a).

Consequently, self‐compassion is considered a form of emotion regulation by some (Diedrich, Grant, Hofmann, Hiller, & Berking, 2014), although others argue the concepts to be complementary (Neff, Kirkpatrick, & Rude, 2007). Acknowledging this discussion, we hypothesize that differentiation between the regulation of positive and negative emotions might provide a valuable perspective. Although the definition of emotion regulation encompasses the regulation of both positive and negative emotions (Gratz & Roemer, 2004), one of the most widely used instruments to assess emotion regulation problems, the Difficulties in Emotion Regulation Scale (DERS), only focuses on the regulation of negative emotions (Weiss, Gratz, & Lavender, 2015). As the general concept of emotion regulation includes the regulation of both negative and positive emotions, a study of the relationship between emotion regulation and mental health should include an instrument capable of assessing the regulation of positive emotions. In the present study, we opted to assess problems with the regulation of negative emotions with the DERS and to add an instrument that also (but not exclusively) covers the positive emotional area.

Regarding the definition of self‐compassion, although Muris, Otgaar, and Petrocchi (2016) argued that the original definition of self‐compassion only includes three positive components (i.e., self‐kindness, common humanity, and mindfulness), Neff (2003a,b, 2016) also includes negative components (i.e., indicators of lack of self‐compassion). However, indicators of a lack of self‐compassion widely overlap with indicators of mental health problems. For example, isolation, an indicator of lack of self‐compassion (Neff, 2003b,b), may be considered a psychopathological symptom itself. Current instruments to assess self‐compassion, for example, the Self‐Compassion Scale (SCS; Neff, 2003b), include such indicators of lack of self‐compassion, which might lead to increased effect sizes of self‐compassion predicting psychopathology (Muris et al., 2016). Also, robust consensus about the factor structures underlying the SCS is still lacking, for example, an overall self‐compassion score, a three‐factor solution, a bidirectional model summing up positive versus negative components, and a six‐factor solution with all separate subscales, are plausible structures (Neff, 2016). Given the overlap and lack of consensus, Muris et al. (2016) recommended the six‐factor solution. However, after failing to replicate the six‐factor structure, López et al. (2015) emphasized it is important to make a distinction between positively and negatively formulated items.

Empirically, the positive components of self‐compassion are confirmed to be protective from psychopathology, albeit not as strong as negative components predict psychopathology (Muris & Petrocchi, 2017). Recognizing the above, we hypothesize that (dys)regulation of negative emotions and self‐compassion together contribute to psychological healthiness. However, it is unclear whether dysregulation of negative emotions and self‐compassion should be expected to provide unique contributions (Diedrich et al., 2014; Neff et al. 2007).

### 1.3 Present study

Based on the above, we formulate two hypotheses: first, EMSs will be positively associated with negative emotion dysregulation and psychopathology and negatively associated with self‐compassion and life satisfaction; Second, the combination of difficulties in negative emotion regulation and self‐compassion mediates the relationship between EMSs, more specifically the EMSs of the D/R domain, and psychopathological symptoms, on the one hand, and satisfaction with life on the other hand. Also, although mutually exclusive at the state level, positive and negative affect dimensions are independent, unipolar structures (Schmukle, Egloff, & Burns, 2002). Thus, we will explore the unique contributions of self‐compassion and difficulties in negative emotion regulation in the relationship between EMS and psychopathology and satisfaction with life.

## 2 METHODS

### 2.1 Sample

Data were collected from a middle‐to‐high income community sample of 296 adult Turkish participants using convenience methods and snowball sampling. The sample consisted of 117 (39.5%) males and 179 (60.5%) females between the ages of 17 and 52 (*M* = 26.85, *SD* = 7.07).

### 2.2 Measurements

The Young Schema Questionnaire—Short Form 3 (YSQ‐S3; Young, Pascal, & Cousineau, 2005) is a 90‐item questionnaire that assesses 18 EMSs that can be grouped into five domains (Young, 1990/1990). Each domain is assessed according to five items (i.e., statements such as “I haven’t had someone to nurture me, share him/herself with me, or care deeply about everything that happens to me.”) scored on a 6‐point scale ranging from 1 (*completely untrue of me*) to 6 (*describes me perfectly*). The YSQ‐S3 has acceptable levels of overall reliability and validity, and for Turkish adolescents and adults specifically (Saritaş & Gençöz, 2011; Soygüt et al., 2009). The Turkish adaptation study revealed adequate internal consistency ranging from *α* = 0.53 (Unrelenting Standards) to *α* = 0.81 (Impaired Autonomy; Saritaş & Gençöz, 2011). In the present study, the α coefficients of the schema domains were found to be ranging from *α* = 0.73 (Impaired Limits) to *α* = 0.90 (Disconnection/Rejection), with a total scale *α* coefficient of 0.96.

The DERS (Gratz & Roemer, 2004) is a 36‐item (e.g., “I have difficulty making sense out of my feelings,” “I have no idea how I am feeling”) self‐report instrument using 5‐point Likert scales (1 = *almost never*, 5 = *almost always*) to asses problems regarding (negative) emotion regulation. The DERS yields a total score as well as five subscores reflecting nonacceptance of emotional responses, difficulty in engaging in the goal‐directed behavior, impulse control difficulties, lack of emotional awareness, limited access to emotion regulation strategies, and lack of emotional clarity, for which higher scores indicate more emotion regulation difficulties. The Turkish translation of the DERS revealed acceptable reliability and validity (Rugancı & Gençöz, 2010). The internal consistency of the total score was found to be 0.94, and the *α* coefficients of the subscales were found to be ranging from *α* = 0.75 (Awareness) to *α* = 0.90 (Impulse). In the present study, *α* coefficients of the subscales were found to be ranging from *α* = 0.69 (Awareness) to *α* = 0.90 (Strategies) for the subscales to *α* = 0.92 for the whole scale.

The SCS (Neff, 2003b) is a 26‐item (e.g., “I try to be loving towards myself when I am feeling emotional pain”) self‐report instrument using 5‐point Likert scales (1 = *almost never*, 5 = *almost always*) to evaluate self‐compassion. The SCS yields total score and six subscale‐scores reflecting three positive subscales (i.e., self‐kindness, common humanity, and mindfulness), and three negative subscales (i.e., self‐judgment, isolation, and over‐identification). The internal consistency of the original scale was found to be *α* = 0.92, and the test–retest reliability was found to be 0.93 for the original scale. The Turkish translation and adaptation of the SCS reported internal consistencies ranging from *α* = 0.69 (Self‐judgement) to *α* = 0.85 (Self‐kindness; Kantaş, 2013); whereas in the present study, the internal consistency of the subscales were found to be ranging from *α* = 0.79 (Mindfulness) to *α* = 0.85 (Self‐judgement).

The Brief Symptom Inventory (BSI, Derogatis, 1975) is the shortened version of the Symptom Checklist (SCL‐90‐R) assessing psychopathological symptoms during the past 7 days based on an individual’s response to 53 items (e.g., feeling fearful, mind going blank, etc.) using 5‐point Likert scales (0 = *not at all*, 4 = *extremely*). The checklist covers nine symptom dimensions, that is, Somatization, Obsession‐Compulsion, Interpersonal Sensitivity, Depression, Anxiety, Hostility, Phobic Anxiety, Paranoid Ideation, and Psychoticism, reflecting three global indices (i.e., global severity index, positive symptom total, and positive symptom distress index). The internal consistencies of the dimensions of the original scale were found to range from *α* = 0.71 (Psychoticism) to *α* = 0.85 (Depression). The test–retest reliability of the subscales was found to range from 0.68 (Somatization) to 0.91 (Phobic Anxiety; Derogatis, 1975). The Turkish translation used in the current study previously revealed acceptable levels of reliability and validity (Şahin & Durak, 1994). In the present study, internal consistency of the whole scale was *α* = 0.97, with subscale consistencies ranging from *α* = 0.79 (Hostility) to *α* = 0.92 (Depression).

The Satisfaction with Life Scale (SWLS; Diener, Emmons, Larsen, & Griffin, 1985) is a 5‐item self‐report instrument using 7‐point Likert scales (1 = *strongly disagree*, 7 = *strongly agree*). The scale yields a total score, for which scores between 0 and 20 indicate the level of dissatisfaction (i.e., lower scores indicate more dissatisfaction with life), whereas scores above 20 indicate the level of satisfaction (i.e., higher scores indicate more satisfaction with life). The internal consistency of the original scale was found to be *α* = 0.87, whereas the test–retest reliability correlation was found to be 0.82 (Diener et al., 1985). Acceptable reliability and validity were reported for the Turkish translation of the SWLS (Durak, Senol‐Durak, & Gencoz, 2010). In the present study, the internal consistency of the scale was found to be *α* = 0.86.

### 2.3 Procedure

The scales were uploaded to an online survey site, and the participants were provided with a link to reach to the questionnaire. Snowball sampling procedure was utilized to reach out to participants who were enthusiastic to participate in the study without being compensated. Individuals were asked to post the link of the study on social media to boost the number of respondents. Caution is warranted, as this may have resulted in a bias (e.g., volunteerism, the absence of random sampling). After giving online consent to participate, participants filled out an online survey consisting of the scales mentioned above. After the completion of the survey, the participants were debriefed about the study and thanked for participation. This procedure was approved by the Applied Ethics Research Center of Middle East Technical University in Ankara, Turkey.

### 2.4 Statistical analysis

Analyses were performed using IBM SPSS Statistics for Windows, Version 23.0 (IBM Corp, Armonk, NY) via Hayes’ (2013) PROCESS procedure for SPSS (Release 2.16.3). PROCESS is a regression‐based approach to model a path analysis of observed variables to estimate direct and indirect effects of the mediation models (Hayes, 2013). We test whether difficulties in negative emotion regulation (i.e., scores on the DERS) and self‐compassion (i.e., the total score on the SCS) mediated the link between the EMSs of the D/R domain, as assessed using the YSQ‐S3, and psychopathological symptoms (i.e., assessed using the BSI) and life satisfaction (i.e., scores on the SWLS). To control for possible effects of other schema domains, that is, the Impaired Autonomy (IA), Other‐directedness (OD), Impaired Limits (IL), and Overvigilance and Inhibition (O/I), these were included to the mediation equation as covariates. Also, age and gender were initially included in the mediation analyses as covariates as they are significantly correlated with the outcome variables. Nonsignificant covariates were deleted from the final model. Mean effects and confidence intervals for scores on the DERS and SCS were estimated applying Hayes’ (2013) bootstrapping procedure with 5,000 resamples. Summed scores were utilized to employ a simpler model. A significance level of *p* < 0.05 was adopted for all statistical tests. Additional analyses were done with only the total score on positive subscales of the SCS, as the inclusion of the negative scales has been criticized (see above).

## 3 RESULTS

Descriptive statistics of, and correlations between, gender, age, psychopathological symptoms (i.e., BSI score), self‐compassion (i.e., SCS score), difficulties negative emotion regulation (i.e., DERS score), life satisfaction (i.e., SWLS score), and scores with regard to the D/R, IA, OD, IL, and O/I domains are reported in Table 1. Among schema domains, D/R had the highest correlation with the DERS and SCS. Psychopathological symptoms were significantly correlated with the D/R domain, SCS, and DERS scores. Among schema domains, D/R and I/A were significantly correlated to satisfaction with life.

**Table 1:** Descriptive statistics and correlations between the included measures

	Mean	*SD*	1	2	3	4	5	6	7	8	9	10	11
1. Gender													
2. Age	26.85	7.07	0.174^**^	1									
3. D/R	2.01	0.65	0.106	–0.052	1								
4. IA	1.86	0.66	–0.057	–0.254^**^	0.680^**^	1							
5. OD	2.64	0.74	0.101	–0.096	0.610^**^	0.693^**^	1						
6. IL	2.99	0.79	0.084	–0.107	0.503^**^	0.407^**^	0.483^**^	1					
7. O/I	2.58	0.70	0.042	–0.159^**^	0.653^**^	0.616^**^	0.704^**^	0.577^**^	1				
8. BSI	.73	0.62	–0.077	–0.262^**^	0.603^**^	0.578^**^	0.510^**^	0.394^**^	0.539^**^	1			
9. SCS	3.45	0.72	0.131^*^	0.201^**^	–0.580^**^	–0.533^**^	–0.424^**^	–0.316^**^	–0.501^**^	–0.564^**^	1		
10. DERS	2.36	0.53	–0.070	–0.230^**^	0.610^**^	0.581^**^	0.491^**^	0.345^**^	0.545^**^	0.631^**^	–0.780^**^	1	
11. SWLS	4.64	1.37	–0.257^**^	–0.231^**^	–0.293^**^	–0.137^*^	–0.097	–0.097	–0.106	–0.247^**^	0.261^**^	–0.226^**^	1

*Note*. BSI: Brief Symptom Inventory; D/R: disconnection/rejection; DERS: Difficulties in Emotion Regulation Scale; IA: Impaired Autonomy; IL: Impaired Limits; OD: Other‐directedness; O/I: Overvigilance and Inhibition; SCS: Self‐Compassion Scale; SWLS: Satisfaction with Life Scale.

^**^ *p* < 0.001.

^*^ *p* < 0.01.

Subsequently, multivariate mediation models were run to test the effects of D/R score on psychopathological symptoms through DERS and SCS. In the case of significant correlations between gender or age and variables in the mediation model, we included the respective variable (i.e., gender or age) in the mediation model.

### 3.1 Symptoms of psychopathology

**Figure 1:**
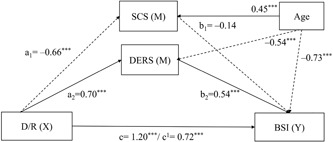
Multiple mediation model of the relationship between D/R, SCS, DERS, and psychopathological symptoms. Arrows represent significant associations. Parameter values are displayed next to respective arrows with the significance level (*p < 0.05; **p < 0.01; ***p < 0.001). Significant positive associations are represented by continuous arrows, whereas negative associations are displayed by dashed arrows. BSI: Brief Symptom Inventory; DERS: Difficulties in Emotion Regulation Scale; D/R: disconnection rejection; SCS: Self‐Compassion Scale

The first mediation model included D/R as the independent variable, BSI as the dependent variable, the two mediators (i.e., SCS and DERS), and all of the possible covariates (i.e., all schema domains other than D/R, age, and gender). In line with the suggestions by Hayes (2013), nonsignificant covariates were excluded from the model, and only age was included in the final multivariate mediation model as a covariate. Results revealed this model (Figure 1 and Table 2) to be significant, *F*(4,291) = 72.62, *p* < 0.001, MSE = 555.54, *r* ^2^ = 0.50, demonstrating that EMS‐D/R domain, SCS, and DERS together explained 50% of the variance in psychopathological symptoms when age was included as a covariate. The bootstrapping analysis with 5,000 resamples for indirect effects revealed a significant total indirect effect (*a*_1_ *b*_1_ + *a*_2_ *b*_2_ = 0.47, *SE* = 0.08, 95% BCa CI [0.34, 0.66]). Confirming our hypothesis, this indicates that SCS and DERS together mediated the relationship between schema domains and symptoms of psychopathology.

**Table 2:** Mediation (indirect effects) of the relationship between D/R and psychopathological symptoms

	Standardized coefficients		Bootstrapping (BCa 95% CI)	
	Effect	*SE*	Lower	Upper
Total indirect effects	0.47^*^	0.08	0.34	0.66
SCS	0.09	0.08	–0.07	0.26
DERS	0.38^*^	0.11	0.19	0.63
DERS vs. SCS	–0.29	0.18	–0.67	0.04

*Note*. BCa: bias corrected and accelerated; CI: confidence interval; DERS: Difficulties in Emotion Regulation Scale; D/R: disconnection/rejection; SCS: Self‐Compassion Scale; *SE*: standard error.

^*^ *p* < 0.05.

More specifically, as can be seen in Figure 1, higher scores on D/R predict lower levels of SCS (*a*_1_ = –0.66, *p* < 0.001) and higher levels of DERS (*a*_2_ = 0.70, *p* < 0.001), the last being related to higher levels of psychopathological symptoms (*b*_2_ = 0.54, *p* < 0.001). Only DERS had a significant mediating effect on the relation between the D/R schema domain and symptoms of psychopathology (*a*_2_ *b*_2_ = 0.38, *SE* = 0.11, 95% BCa CI [0.19, 0.63]). SCS did not contribute to the indirect effect (Table 2). In addition to the total mediation effect (*c* = 1.20, *p* < 0.001), the D/R score had a significant direct effect (*c*_1_ = 0.72, *p* < 0.001) on symptoms of psychopathology (Figure 1). Hence, our results corroborate the hypothesis that the D/R domain is positively associated with DERS and psychopathological symptoms; whereas it is negatively associated with SCS.

### 3.2 Life satisfaction

The second model we applied tested the effects of the D/R domain in combination with DERS and SCS on life satisfaction. To control for possible effects of age, gender, and other schema domains, these were initially included in the mediation equation as covariates.

Results demonstrated a significant model, *F*(5,290) = 16.95, *p* < 0.001, MSE = 36.90, *r* ^2^ = 0.23, indicating D/R, SCS, and DERS together explaining 23% of the variance in satisfaction with life when controlling for age and gender. The bootstrapping analysis with 5,000 resamples for indirect effects revealed a significant total indirect effect (*a*_1_ *b*_1_ + *a*_2_ *b*_2_ = –0.07, *SE* = 0.02, 95% BCa CI [–0.11, –0.03], *p* < 0.001). However, DERS did not have a significant mediating effect in the relationship between D/R and life satisfaction, whereas SCS was significant as mediator (*a*_1_ *b*_1_ = –0.06, *SE* = 0.02, 95% BCa CI [–0.11, –0.02]; Table 3).

**Table 3:** Mediation (indirect effects) of the relationship between D/R and satisfaction with life

	Standardized coefficients		Bootstrapping (BCa 95% CI)	
	Effect	*SE*	Lower	Upper
Total indirect effects	–0.07^*^	0.02	–0.11	–0.03
DERS	–0.01	0.02	–0.05	0.03
SCS	–0.06^*^	0.02	–0.11	–0.02
DERS vs. SCS	–0.05	0.04	–0.13	0.03

*Note*. BCa: bias corrected and accelerated; CI: confidence interval; DERS: Difficulties in Emotion Regulation Scale; D/R: disconnection/rejection; SCS: Self‐Compassion Scale; *SE*: standard error.

^*^ *p* < 0.05.

**Figure 2:**
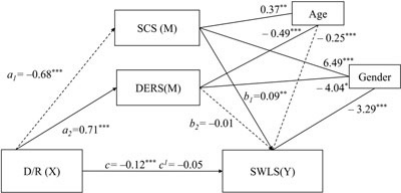
Multiple mediation model of the relationship between D/R, SCS, DERS, and life satisfaction. Arrows represent significant associations. Parameter values are displayed next to respective arrows with the significance level (*p < 0.05; **p < 0.01; ***p < 0.001). Significant positive associations are represented by continuous arrows, whereas negative associations are displayed by dashed arrows. DERS: Difficulties in Emotion Regulation Scale; D/R: disconnection/rejection; SCS: Self‐Compassion Scale; SWLS: Satisfaction with Life Scale

More specifically, results of our (Figure 2 and Table 3) model revealed a direct negative effect of D/R on SCS (*a*_1_ = –0.68, *p* < 0.001) and a direct positive effect on DERS (*a*_2_ = 0.71, *p* < 0.001). SCS had a positive direct effect on satisfaction with life (*b*_1_ = 0.09, *p* < 0.01), whereas DERS did not (*b*_2_ = –0.01, *p* > 0.05). The total effect of D/R on life satisfaction was significant (*c* = –0.12, *p* < 0.001), whereas the direct effect was not (*c*_1_ = –0.05, *p* > 0.05). Thus, as hypothesized, the D/R domain was negatively associated with life satisfaction, and DERS and SCS together mediated the relationship between the D/R domain and life satisfaction.

### 3.3 Additional analyses: Positive components of self‐compassion

Taking into consideration recent concerns regarding the use of the SCS total score (i.e., as a function of positive and negative dimensions) expressed by Muris et al. (2016), we further analyzed the results by including only positive dimensions of the SCS, revealing similar results.

### 3.4 Positive components of self‐compassion and psychopathological symptoms

**Figure 3:**
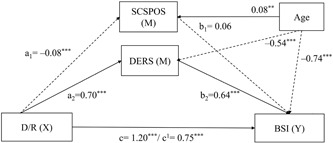
Multiple mediation model of the relationship between D/R, positive components of SCS, and psychopathological symptoms. Arrows represent significant associations. Parameter values are displayed next to respective arrows with the significance level (*p < 0.05; **p < 0.01; ***p < 0.001). Significant positive associations are represented by continuous arrows, whereas negative associations are displayed by dashed arrows. BSI: Brief Symptom Inventory; DERS: Difficulties in Emotion Regulation Scale; D/R: disconnection/rejection; SCSPOS: Self‐Compassion Scale Positive Dimensions

As depicted in Figure 3, the third model, *F*(4,291) = 71.93, *p* < 0.001, MSE = 558.20, *r* ^2^ = 0.50, revealed that the D/R still had a direct negative effect on positive components of SCS (*a*_1_ = –0.08, *p* < 0.001), however, the coefficient was considerably smaller than the coefficient in the first model (*a*_1 = _–0.66, *p* < 0.001), depicted in Figure 1. Comparable to the first model, positive components of SCS showed no effects on symptoms of psychopathology (*b*_1_ = 0.06, *p* > 0.05).

The bootstrapping analysis with 5,000 resamples for indirect effects revealed a significant total indirect effect (Table 4). In terms of mediators, only DERS had a significant mediating effect on the relationship between schema domains and symptoms of psychopathology (*a*_2_ *b*_2_ = 0.45, *p* < 0.05). SCS did not contribute to the indirect effect beyond DERS. Different from the original results, investigation of pairwise contrasts of the indirect effects of the mediators revealed that the specific indirect effects differed significantly from each other (*p* < 0.05).

**Table 4:** Mediation (indirect effects) of the relationship between D/R and psychopathological symptoms based on positive dimensions of the SCS

	Standardized coefficients		Bootstrapping (BCa 95% CI)	
	Effect	*SE*	Lower	Upper
Total indirect effects	0.44^*^	0.08	0.30	0.61
SCSPOS	–0.005	0.04	–0.09	0.09
DERS	0.45^*^	0.10	0.27	0.66
DERS vs. SCSPOS	–0.45^*^	0.13	–0.72	–0.21

*Note*. CI: confidence interval; BCa: bias corrected and accelerated; DERS: Difficulties in Emotion Regulation Scale; D/R: disconnection/rejection; SCSPOS: Self‐Compassion Scale Positive Dimensions; *SE*: standard error.

^*^ *p* < 0.05.

### 3.5 Positive components of self‐compassion and life satisfaction

**Figure 4:**
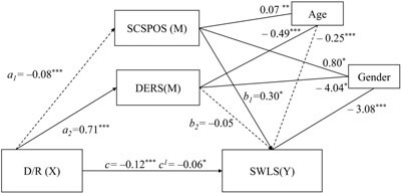
Multiple mediation model of the relationship between D/R, positive components of SCS, DERS, and life satisfaction. Arrows represent significant associations. Parameter values are displayed next to respective arrows with the significance level (*p < 0.05; **p < 0.01; ***p < 0.001). Significant linear trends are represented by nondashed arrows, whereas negative associations are displayed by dashed arrows. DERS: Difficulties in Emotion Regulation Scale; D/R: disconnection/rejection; SCSPOS: Self‐Compassion Scale Positive Dimensions; SWLS: Satisfaction With Life Scale

The fourth multivariate mediation model, *F*(5,290) = 16.27, *p* < 0.001, MSE = 37.24, *r* ^2^ = 0.22, revealed results similar to the second model (Figure 4). D/R still demonstrated a direct effect on positive dimensions of SCS (*a*_1_ = –0.08, *p* < 0.001), although considerably smaller than the model depicted in Figure 2 (*a*_1_ = –0.68, *p* < 0.001). Positive dimensions of SCS had a stronger positive direct effect on satisfaction with life (*b*_1_ = 0.30, *p* < 0.001) compared with the original model (*b*_1_ = 0.09, *p* < 0.01), whereas results of DERS (*b*_2_) did not change. Different from the original model, both direct (*c*) and indirect (*c*_1_) effects of the D/R on life satisfaction were significant (*c*).

The bootstrapping analysis with 5,000 resamples for indirect effects revealed a significant total indirect effect (*a*_1_ *b*_1_ + *a*_2_ *b*_2_ = –0.06, *SE* = 0.02, 95% BCa CI [–0.10, –0.02]). Similar to the original model, only self‐compassion (*a*_1_ *b*_1_ = –0.03, *SE* = 0.01, 95% BCa CI [–0.05, –0.004]) had a significant mediating effect between D/R and satisfaction with life (Table 5). However, the difference between SCPOS and DERS in mediating effect failed to reach significance (Table 5).

**Table 5:** Mediation (indirect effects) of the relationship between D/R and satisfaction with life based on positive dimensions of the SCS

	Standardized coefficients		Bootstrapping (BCa 95% CI)	
	Effect	*SE*	Lower	Upper
Total indirect effects	–0.06^*^	0.02	–0.10	–0.02
DERS	–0.03	0.02	–0.07	0.002
SCSPOS	–0.03^*^	0.01	–0.05	–0.004
DERS vs. SCSPOS	0.007	0.03	–0.04	0.06

*Note*. BCa: bias corrected and accelerated; CI: confidence interval; DERS: Difficulties in Emotion Regulation Scale; D/R: disconnection/rejection; SCS: Self‐Compassion Scale; SCSPOS: Self‐Compassion Scale Positive Dimensions; *SE*: standard error.

^*^ *p* < 0.05.

## 4 DISCUSSION

Mental health comprises the presence of positive emotions and the ability to regulate negative emotions (Keyes, 2005; Westerhof & Keyes, 2010); both positive and negative affective dimensions are important to consider (Kahl et al., 2012). Previous research indicates the presence of EMSs is associated with psychopathology and lower levels of life satisfaction (Young et al., 2003). EMSs are often triggered unconsciously, which makes it hard for a person to fully realize in which ways EMSs affect daily life, let alone control the activation of such EMSs that leads to strong negative emotions (Bernstein, 2005; Young et al., 2003). Emotion regulation strategies are used automatically to deal with the emotional distress caused by EMSs. Hence, dysfunctions in regulating positive or negative emotions possibly mediate the relation between EMSs, psychopathology, and life satisfaction. The present study aimed to get a better understanding of problems in regulating negative emotions and self‐compassion in the relationship between EMSs and psychopathological symptoms, respectively life satisfaction. We hypothesized that, of all EMSs, especially EMSs in the D/R domain positively contribute to symptoms of psychopathology and lower levels of life satisfaction, and that these contributions may be mediated by difficulties in negative emotion regulation and self‐compassion. We additionally explored the unique contribution of both difficulties in negative emotion regulation and self‐compassion.

First of all, as predicted, results of an initial correlation analysis revealed that, of all schema domains (Young et al., 2003), the D/R domain had relatively stronger correlations with self‐compassion, difficulties in negative emotion regulation, symptoms psychopathology, and life satisfaction. In addition, the strong correlation between negative emotion regulation difficulties and self‐compassion seems to support the idea that the two widely overlap (Diedrich et al., 2014). However, subsequent multiple mediation models demonstrated that negative emotion regulation difficulties and self‐compassion mediated the relationship between EMSs in the D/R domain on the one hand, and psychopathology and life satisfaction on the other hand, in different ways. That is, EMSs in the D/R domain predicted both symptoms of psychopathology and life satisfaction; whereas the former relation (i.e., EMSs in the D/R domain—symptoms of psychopathology) was mediated by negative emotion regulation difficulties, the latter relation (i.e., D/R domain EMSs—life satisfaction) was mediated by self‐compassion. More specifically, regarding mediation effects, difficulties in negative emotion regulation partially mediated the relationship between EMSs D/R domain and psychopathological symptoms; the direct effect of D/R EMSs on psychopathological symptoms remained significant after controlling for this mediation effect. The effect of D/R EMSs on life satisfaction, on the other hand, disappeared when controlling for self‐compassion, which fully mediated the relationship between D/R domain EMSs and life satisfaction.

A possible explanation for these findings might be found in the differential effects of positive and negative emotions and strategies. That is, psychopathological symptoms are associated with the presence of negative affect, and emotion dysregulation signals a failure to regulate negative emotions (Fassbinder et al., 2016). On the contrary, in addition to three negative dimensions, self‐compassion includes three positive dimensions (Neff, 2003a,b). These positive dimensions reflect positive attitudes toward oneself that help to attain a positive emotional state, which is, in turn, associated with life satisfaction. Indeed, our results show that the mediating effect of self‐compassion, although decreased in magnitude, remained when including only the positive dimensions of the self‐compassion scale.

Our findings underline the need for a better understanding of the difference and overlap between negative emotion regulation and self‐compassion; the literature provides no consensus about the question whether self‐compassion is an emotion regulation technique or a complimentary strategy (Diedrich et al., 2014; Neff et al., 2007). In the present study, despite a strong correlation between emotion regulation difficulties and self‐compassion, self‐compassion and negative emotion regulation difficulties demonstrated unique, differential effects. The particular importance of self‐compassion regarding life satisfaction underlines the previously emphasized connection between self‐compassion and positive emotion regulation (Neff, 2003b; Neff et al., 2007). In addition, our results suggest possible differential roles of positive and negative emotion regulation in mental health, but developing a full picture, especially regarding the role of positive emotion regulation, needs further inquiry. Therefore, more direct measurement tools for positive emotion regulation (e.g., DERS‐positive), which were not available at the time of our data collection, are recommended for future research.

Next to that, our findings support the idea that addressing EMSs only regarding psychopathological symptoms and negative emotions limits its treatment potential. That is, in improving coping with EMSs it, may be recommended to focus both on enhancing positive emotions, and on decreasing negative emotions. The term emotion dysregulation is often used to describe problems with regulating negative emotions. Positive emotions and the disability to attain them are often overlooked; a disability to create positive emotional states is generally not labeled as emotion dysregulation. Self‐compassion appears to be important for the presence of easily overlooked positive affect. However, whereas the connection between schema therapy and dysregulation of negative emotions has been studied extensively (Dadomo et al., 2016), the effectiveness of schema therapy including the regulation of positive emotions and increasing positive emotional experiences needs further inquiry. In this regard, the integration of schema therapy elements in combination with contemporary positive psychology practices like acceptance, mindfulness, and self‐compassion provides an interesting research area with promising clinical potential.

Some limitations should be kept in mind when interpreting the results of the present study. First, the sample is nonclinical, which means that the conclusions from the present study may not be generalizable to clinical populations. Second, the cross‐sectional design prohibits inferring time‐dependent relationships. Third, the results do not imply causation between the variables; the mediating roles of difficulties in negative emotion regulation and self‐compassion need experimental confirmation. Fourth, when looking at the results about life satisfaction, the effect sizes were small. One possible reason for this might be that the instrument used only assesses part of the life satisfaction construct. Including direct indicators of positive affect might result in stronger effect sizes and more reliable conclusions in future research.

## 5 CONCLUSION

To conclude, we demonstrated that the D/R domain remains the most important EMS domain regarding psychopathological symptoms and life satisfaction. Also, self‐compassion mediates the relationship between EMSs of the D/R EMSs and life satisfaction, with no additional role for negative emotion dysregulation, whereas negative emotion dysregulation mediates the relationship between D/R EMSs and psychopathological symptoms, with no additional contribution of self‐compassion. These conclusions remain when taking into account only positive components of self‐compassion. This indicates that self‐compassion and negative emotion regulation represent complementary concepts, each playing a unique role in different aspects of mental health.
